# Effects of Charged Particles on Human Tumor Cells

**DOI:** 10.3389/fonc.2016.00023

**Published:** 2016-02-12

**Authors:** Kathryn D. Held, Hidemasa Kawamura, Takuya Kaminuma, Athena Evalour S. Paz, Yukari Yoshida, Qi Liu, Henning Willers, Akihisa Takahashi

**Affiliations:** ^1^Department of Radiation Oncology, Massachusetts General Hospital, Harvard Medical School, Boston, MA, USA; ^2^Gunma University Heavy Ion Medical Center, Gunma, Japan; ^3^Department of Radiation Oncology, Gunma University Graduate School of Medicine, Gunma, Japan

**Keywords:** charged particles, proton therapy, carbon-ion therapy, relative biological effectiveness, clustered DNA damage, cancer stem cells, hypoxic radioresistance, altered fractionation

## Abstract

The use of charged particle therapy in cancer treatment is growing rapidly, in large part because the exquisite dose localization of charged particles allows for higher radiation doses to be given to tumor tissue while normal tissues are exposed to lower doses and decreased volumes of normal tissues are irradiated. In addition, charged particles heavier than protons have substantial potential clinical advantages because of their additional biological effects, including greater cell killing effectiveness, decreased radiation resistance of hypoxic cells in tumors, and reduced cell cycle dependence of radiation response. These biological advantages depend on many factors, such as endpoint, cell or tissue type, dose, dose rate or fractionation, charged particle type and energy, and oxygen concentration. This review summarizes the unique biological advantages of charged particle therapy and highlights recent research and areas of particular research needs, such as quantification of relative biological effectiveness (RBE) for various tumor types and radiation qualities, role of genetic background of tumor cells in determining response to charged particles, sensitivity of cancer stem-like cells to charged particles, role of charged particles in tumors with hypoxic fractions, and importance of fractionation, including use of hypofractionation, with charged particles.

## Introduction

Radiation therapy is a mainstay of cancer treatment, being a common and effective therapy for both curative and palliative treatment of cancer patients. In the last few decades, there has been increasing use of charged particles in radiation therapy. Protons were first proposed for use in cancer therapy by Robert R. Wilson (1), and the number of patients treated with protons has increased dramatically in recent years to a total of over 100,000 patients now treated worldwide (http://www.ptcog.ch/index.php/facilities-in-operation). Radiation treatment of cancer with helium ions began at Berkeley in the late 1950s and was expanded to heavier ions in the 1970s [see a review of the history of charged particles by Skarsgard (2)]. Much of the emphasis has been on carbon ions, with most patients treated in Japan and now totaling over 10,000 patients treated worldwide. The major clinical advantage of protons and heavier charged particles, such as carbon, comes from physics: the Bragg curve provides excellent radiation dose distributions [see reviews in Ref. (3, 4)]. In addition, heavier ions, e.g., carbon, offer the potential of additional biological gains such as increased relative biological effectiveness (RBE) and decreased oxygen enhancement ratio (OER) due to their higher linear energy transfer (LET) in the Bragg peak region, where the tumor is located [reviewed, e.g., in Ref. (3, 5, 6)].

Despite the often-made assumption that the RBE for tumor cells is higher than that for normal cells irradiated under identical conditions, there is only a limited amount of experimental *in vitro* data that support that assertion (3). However, there have been interesting recent research findings on the differential DNA repair pathways of cancer cells after particle versus photon irradiation, new studies on the effects of charged particles on cancer stem cells, and increasing questions about different responses of tumor and normal cells to hypofractionation, especially with charged particle irradiations, suggest that there may be novel ways to take advantage of differences in characteristics of tumor cells from normal cells to improve or better tailor the use of charged particles in cancer therapy. This review will discuss these issues, with emphasis on data on responses of human tumor cells, largely based on *in vitro* findings. As discussed in more detail below, RBE is a complex quantity, depending on physical parameters, such as particle type and energy, dose and LET, and biological parameters, including cell/tissue type, cell cycle phase, oxygen level, and endpoint. *In vitro* assays have limitations compared to *in vivo* studies and the clinical situation due to lack of 3D architecture and microenvironmental context, including interactions among various cell types, vasculature, and immune system influences. Nevertheless, for studies of RBE, *in vitro* assays are critical for systematic testing and characterization of effects of various ions, elucidation of DNA damage pathways, and the importance of DNA repair processes and other genetic factors. Furthermore, *in vitro* studies provide experimental tests for validation of biophysical models, e.g., the local effects model (LEM), prior to clinical application (7), and yield insight on systematic variations in RBE relevant to clinical use (8, 9).

In this review, we start with brief overview sections on the unique biological advantages of charged particle therapy and DNA damage responses that may be important for particle therapy. That introduction is followed by consideration of recent findings on RBEs in human tumor cells, including discussion of the possible roles of genetic factors on RBE, then discussions of new findings on cancer stem cells, hypoxia, and fractionation. In particular, we stress approaches to use the increasing knowledge of the properties of tumors and tumor cells to better advantage when using charged particles in cancer therapy.

## An Overview of the Unique Biological Advantages of Charged Particle Therapy

A number of reviews [e.g., in Ref. (3–5)] have discussed the substantial dose distribution advantages of charged particles where, as a result of the Bragg peak, normal tissues can be spared by limiting dose to them, while maximum dose is deposited in the tumor. Heavier ions, such as carbon, have an additional dose distribution advantage over protons because of their reduced lateral scattering compared to protons. However, the major potential advantage of heavier ions in tumor irradiations is their enhanced biological effects, which include increased cell killing, decreased protection by hypoxia, decreased effect of fractionation, and decreased cell cycle dependence. The biological effectiveness of cell killing by higher LET radiations is usually quantified by use of RBE, the ratio of the dose of low-LET radiation (usually X-rays or gamma-rays) to dose of high-LET radiation (e.g., charged particle) for the same biological effect. Many *in vitro* studies over the years have shown the bell-shaped dependence of RBE for cell killing on LET (6, 10–12) wherein RBE increases with LET to a maximum at about 30–150 keV/μm, then decreases at higher LET. The LET value at which the RBE is maximal depends on the individual ion species, with the peak at higher LET with increasing atomic number of the ions (2). Furthermore, it has also long been recognized that there is great variation in the absolute values of RBE because RBE depends on numerous factors, including particle type and energy, cell type, experimental endpoint, cell cycle phase, dose and dose rate, oxygenation status, culture conditions, etc. (6, 7, 11).

The increased biological effectiveness of radiations with increasing LET lies in the physical dose distribution of the energy of the particles on the micro, and even nano, scale as they traverse matter, the clustering of DNA damages that results from the particle tracks and the increased difficulty cells have in accurately repairing the clustered damage (13–16). As energetic charged particles traverse matter, e.g., cells and tissues of organisms, their electronic interactions with atoms and molecules, mostly through inelastic collisions with atomic electrons, create a path, or track of ionizations before they run out of energy at a finite range, the Bragg peak. The tracks of heavy charged particles are fairly straight, but the electrons ejected from atoms along the track, being much lighter, follow paths that are quite tortuous, with their ranges depend on the energy they acquired when ejected. LET is a measure of the energy imparted to matter by the passage of an ionizing particle. Along the path of a charged particle, the three-dimensional distribution of energy depositions, which cause ionizations and excitations, is called the track structure. For low-LET sparsely ionizing radiations, there are relatively long distances between the energy depositions except at track ends, but with increasing LET, the ionizations along the track become denser and there is lateral spread of the track due to delta-ray electrons, the spectrum of which is determined by the velocity of the heavy charged particle.

If the ionizations from radiation were randomly distributed in cells, the consequences of those energy depositions would likely be minimal, but the non-randomness of the energy depositions accounts for the increased effectiveness of ionizing radiation (14, 17, 18). The clustering of ionizations along radiation tracks occurs on the same scale as the diameter of a DNA molecule and nucleosomes such that if a track traverses DNA it can effectively create clustered DNA damages, such as double-strand breaks (DSBs), clusters of two or more base damages, or clusters of single-strand breaks with base damages. As LET of radiation increases, the clustering becomes more complex, creating, for example, a complex DSB where the break is associated with additional damages, such as base changes or single-strand breaks. Both the proportion and degree of complexity increase with high-LET radiations (19). A number of studies have shown that the complex DNA damages produced by high-LET radiations are repaired less rapidly, less accurately, and less completely than damages from low-LET photons [reviewed recently in Ref. (20, 21)]. Additionally, it is important to bear in mind that track structure has biological relevance not only at the level of DNA damage but also at higher levels of chromatin organization (17): a single high-LET particle track passing through a cell nucleus may cause correlated damages through chromatin structures, such as chromatin fibers, or in adjacent chromosome territories *via* a string of DSBs along its path, and these correlated damages may result in complex chromosome aberrations. Altogether, the net effect is that complex DNA damages resulting from the greater clustering of ionizations with increasing LET of radiation increases the production of all chromosome aberrations, simple as well as complex.

The increased DNA damage complexity and decreased repair accuracy with radiations of increasing LET not only cause increased cell killing but also result in decreased cell cycle dependence of that killing and play a factor in the decrease in OER. Cells exposed to low-LET radiation show increased resistance when irradiated in late S-phase and increased sensitivity when irradiated in M-phase (22). This fluctuation through the cell cycle decreases with higher LET radiations. However, since in many tumors, the majority of cells are not in the radiation-resistant phases, this effect on treatment outcome in irradiated tumors is likely to be modest (3). The importance of the decreased OER with high LET is discussed below.

Although there has been increasing interest in recent years in the so-called “non-targeted” effects of radiation, including bystander effects and genomic instability in progeny of irradiated cells [for recent reviews, see Ref. (23, 24)], it remains far from clear whether non-targeted effects are similar or different after irradiation with photons versus charged particles (25–27). Furthermore, the role of non-targeted effects or intercellular signaling in response of tumors to radiation remains under investigation (28, 29), with very little work having been done with charged particles. This review is limited to discussion of targeted effects of charge particles.

## Overview of DNA Damage Responses Relevant to Charged Particle Biology

Central to any consideration of the effects of charged particles on cells and tissues must be DNA damage response processes. Cells have two main pathways for the repair of radiation-induced DSBs: non-homologous end-joining (NHEJ) and homologous recombination (HR) (30–32). NHEJ is active throughout the cell cycle and is responsible for the repair of most DSBs in cells. NHEJ involves the initial binding of the Ku70/Ku80 heterodimer, recruitment of DNA–PKcs and eventual ligation of the DNA ends by XRCC4–DNA Ligase IV. However, NHEJ is an error-prone repair, and the quality of its repair processes can decrease with increasing levels of DNA damage. HR is active primarily during the S/G2 phases of the cell cycle, when a homologous DNA region is available, and generally results in the preservation of the original DNA sequence. HR involves DSB recognition by the MRN complex (Mre11, Rad50, Nbs1), 3′–5′ DNA resection, DNA stabilization by replication protein A (RPA), Rad51-mediated formation of Holliday junctions, and ultimately resolution of the Holliday junction (31, 33). HR is also involved in the repair and restart of collapsed DNA replication forks (34). At the forks, the BRCA1/2-dependent HR pathway converges with the Fanconi anemia (FA) pathway to resolve the damage (35). It has been suggested that unrepaired clustered DNA damages that collide with replication forks in cells in S-phase require HR for DNA repair and replication restart (36, 37).

It also has been reported that the end-resection activity in cells in the G_1_ phase may promote micro-homology-mediated end joining (MMEJ) to repair DSBs that cannot be repaired efficiently by NHEJ (38). However, it is unknown how much the activation of HR and MMEJ pathways contribute to escaping cell death in high-LET-irradiated cells. Recently, we showed that targeting and suppressing NHEJ repair yields a high radiosensitivity in cells exposed to carbon-ion beams when compared to the suppression of HR repair (39).

## RBEs of Charged Particles in Human Tumor Cells

Experimental studies to determine RBEs have been conducted for many years, with the majority using clonogenic cell survival as the endpoint. It has been felt that lack of clonogenicity is a highly relevant indicator of the efficacy of radiation and its modification because eradication of tumor cells is needed to cure tumors (22). In fact, the shape of curves of tumor control probability, as detected in a clinical context, can be explained from the random nature of tumor cell killing by radiation and the need to kill every cell, as a single cell may give rise to tumor regrowth (22). Furthermore, RBE values, measured or predicted by computer models, are used in clinical treatment planning approaches, which are continually being updated [e.g., Ref. (40, 41)].

It has been argued recently that further studies measuring RBE values may be of limited usefulness because they will have little impact on reducing the uncertainties in ion beam therapy (4, 6). However, determinations of RBEs can help guide understanding of mechanistic underpinnings to the increased effectiveness of higher LET radiations and, thus, may lead to better identification, based on genetic profiles or biomarker evaluation, of patients’ tumors that may benefit most from charged particle therapy.

### Shifting the Paradigm of a Generic RBE for Clinical Proton Beam Therapy

Clinical proton beam therapy has been based on the use of a generic RBE of ~1.1 at the center of the spread-out Bragg peak (SOBP) for cancer as well as for normal tissues (8). This RBE value represents an average of a wide range of experimental data *in vitro* and *in vivo* and has been intended to be a conservative estimate (8, 42). However, there is now a growing appreciation that the use of a generic value ignores RBE variations that may result, for example, from the heterogeneity of human cancers, LET variations along the SOBP, or the particular clinical endpoint under consideration (42–46). In this section, we will focus primarily on recent data that indicate a dependence of RBE on certain DNA repair defects, with the implication being that proton therapy may have a biological advantage in human tumors that harbor such defects.

There exists very little experimental data on RBE variations in human cancers. In a 2002 review by Paganetti and colleagues (8), the average RBE at the mid-SOBP was estimated as ~1.2 *in vitro* and ~1.1 *in vivo*. However, most of the 20 cell lines considered in that analysis were of rodent origin resulting in a somewhat higher *in vitro* RBE. Only seven human cancer cell lines were included. There is growing evidence for considerable genomic heterogeneity across cancers even of the same type and histology, and it is increasingly appreciated that much of the variations in treatment sensitivity observed clinically are due to genomic heterogeneity, which may include alterations of DNA repair pathways (47–49). Therefore, it is highly doubtful that small numbers of non-representative cell lines are adequate pre-clinical models for assessing clinically relevant variations in RBE values in human cancers. In a recent screen of 17 lung cancer cell lines, RBE estimates at the mid-SOBP of a clinical beam relative to Co60 photons [Co60 equivalent (Eq)] ranged from 0.93 to 1.77 and 1.09 to 1.48 for clonogenic survival fractions of 0.5 and 0.1, respectively (44). In five cell lines (29%), the RBE increase was statistically different from 1.1. Furthermore, in at least three of these cell lines, the RBE increase correlated with defects in the so-called FA/BRCA pathway of DNA repair, and this observation was confirmed in several isogenic cell line models. The FA/BRCA pathway is critical for the maintenance and repair of DNA replication forks [reviewed in Ref. (34, 50)]. Inactivation of any of the FA/BRCA genes has been known to result in hypersensitivity to a variety of anti-cancer agents. However, apart from an involvement of the RAD51 recombinase (FANCR) in the cellular response to proton radiation (43, 51), the importance of the FA/BRCA genes for the repair of proton damage to DNA had been unknown. These observations are clinically significant because genetic or epigenetic defects in the FA/BRCA pathway have been found in large subsets of human cancers (34).

**Figure 1:**
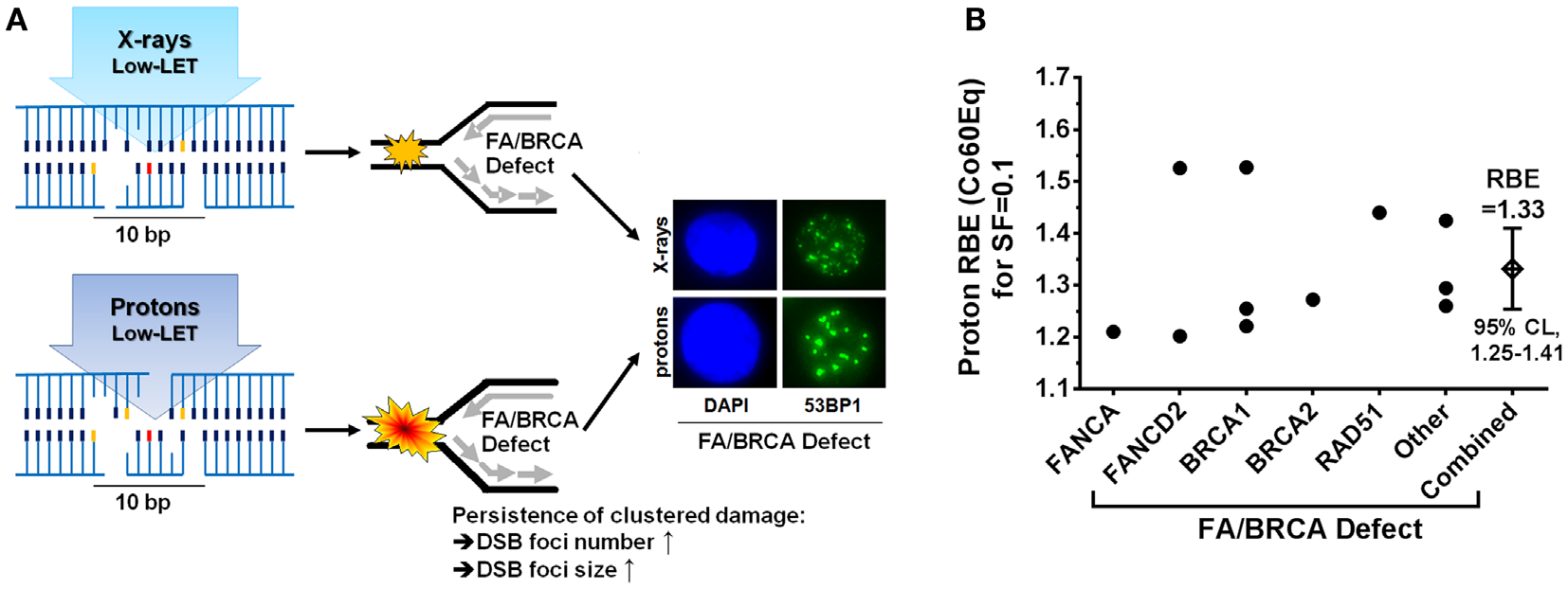
**A “New Biology” of proton beam therapy**. **(A)** Illustration of how FA/BRCA defects may sensitize cells to proton irradiation. Left, clustered DNA damages after equal physical doses of X-rays and mid-SOBP protons are slightly different despite similar LET (2–2.5 keV/μm) and identical RBE in repair-proficient cells (~1.1). In the presence of a FA/BRCA defect that affects the repair of replication forks encountering clustered damages, there will be greater unrepaired damage after proton-irradiation, as marked by an increased number and relative size of repair-related protein accumulations of DNA double-strand break markers. Representative immunofluorescence microscopy images showing nucleus (DAPI) and 53BP1 foci (green) in FANCD2-mutant cells are shown on the right. **(B)** Summary of RBE estimates relative to Co60 photons as a function of defects in the FA/BRCA pathway (44, 45). Other, taken from unpublished data (Willers et al.); CoEq, Co60 equivalent; SF, surviving fraction; CL, confidence limits.

What are the mechanisms through which the FA/BRCA pathway acts on proton damage? For low-LET radiation, which includes X-rays and protons, it has been estimated that 20–40% of the initial damage is clustered, and the majority of clustered damage is present as non-DSB damage (52, 53). Proton radiation causes slightly more complex clustered DNA damages than photons, which is a reflection of the different LET values, i.e., ~2.5 keV/μm for protons at mid-SOBP versus ~0.3–2.0 keV/μm for different photon radiations. DNA repair-proficient tumor cells and normal cells remove these damages almost equally well, consistent with a proton RBE of 1.1 (Co60Eq). Because the FA genes are specifically involved in replication fork maintenance and repair, it can be inferred that the RBE increase that is seen with defects in this pathway results from impaired repair of forks that collide with clustered proton damages. The requirement for the FA/BRCA pathway is greater for proton damage compared to damage caused by, for example, X-rays, even though the RBE (Co60Eq) and LET of these two radiation modalities are almost identical [RBE(Co60) ~1.1 and LET = 2.0–2.5 keV/μm]. This is illustrated in Figure 1A. Proton-irradiated FA/BRCA-defective cells will accumulate greater numbers of DNA DSB in S-phase and subsequently G2-phase than X-irradiated cells, as has been shown experimentally (44) (Willers et al., unpublished). Interestingly, an increase in the size of DSB-associated foci persisting after proton irradiation has been observed (44), likely signifying unrepaired clustered damages (Figure 1A). It has been proposed that these DSB foci could serve as predictive biomarkers to identify cancers that may be more susceptible to proton beam therapy (44). Alternatively, genetic or epigenetic defects in the FA/BRCA pathway could be detected through genomics techniques in order to identify patients for proton therapy. This approach will require a more detailed knowledge of the genes involved in the cellular response to clustered proton damages. The available data indicate that functional loss of any of several key genes in the FA/BRCA pathway will increase the RBE, with the best current estimate being an average RBE of 1.33 (95% confidence limits, 1.25–1.41) at mid-SOBP as shown in Figure 1B. This is a conservative estimate derived at a surviving fraction of 0.1. For 0.5 survival fraction, which is more applicable to fraction sizes of 2 Gy as used in the clinic and which overlaps with the shoulder of the survival curves, the RBE values of the most proton-sensitive cell lines tended to be even higher than for 0.1 survival fraction. For example, the five most sensitive lung cancer cell lines in the report by Liu et al. (44) had an average RBE of 1.30 (range, 1.22–1.48) and 1.46 (range, 1.31–1.77) at survival fractions of 0.1 and 0.5, respectively.

In conclusion, these recent pre-clinical data strongly suggest inter-tumoral heterogeneity of proton RBE that may yield opportunities to identify proton susceptible tumors in the clinic within the next few years. This “New Biology” of protons in cancer coupled with the increasing knowledge of RBE variations as a function of physical proton beam parameters in both cancers and normal tissues is expected to shift the paradigm of a generic proton RBE to a variable RBE.

### RBE Determinations with Heavy Charged Particles

The proton studies just described provide a possible DNA repair capacity-based explanation for some of the variation seen in proton RBE values at a given LET. Could a similar finding apply to human tumors exposed to high-LET charged particles? Unfortunately, no single study with a substantial number of cell lines has yet been done for any heavy ion, although many small studies with a few cell lines each have been performed. Some large compilations of cell survival RBE values for many cell types, endpoints and radiation qualities have been published recently (7, 54, 55), and the composite data clearly show that RBEs depend on LET, endpoint, ion, etc. In this section, we focus on analysis of RBE values for human tumor cells exposed to ions heavier than protons. Published papers that describe the cell survival RBE of human tumor cells have been searched by using PubMed; many of these papers are included in the compilations mentioned. A total of 430 RBE values were collected from 36 published papers (56–91). When authors provided RBE values along with dose–response data, those values were used. In cases where authors showed dose–response curves but did not cite any RBE value, an isoeffect line was drawn in the dose–response curves to read corresponding doses of ions and reference photons. As reference beam, 30 papers used X-rays and 6 papers used gamma-rays. For the analyses here, the biological differences in effect between X-rays and gamma-rays were not considered.

#### Endpoint

**Figure 2:**
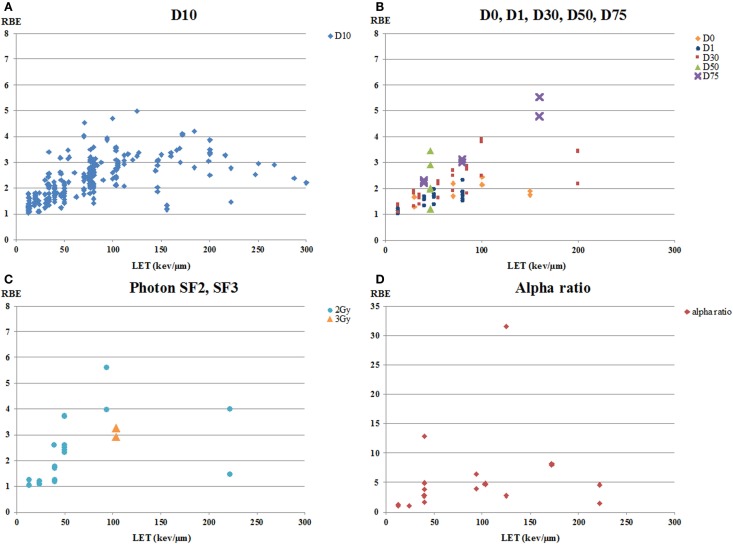
**RBE versus LET for human tumor cell lines for various endpoints**. RBE values are all based on colony formation assays. **(A)** RBE values calculated as the ratio of isoeffect doses at 10% survival (D10). **(B)** RBE values calculated as ratios of doses for D0, D30, D50, and D75. D0 was calculated by fitting the survival curve to the single-hit multi-target (SHMT) model: S/S0 = 1 − (1 − e^−D/D0^)^n^. **(C)** RBE values calculated as the ratio of doses at the level of photon doses of 2 Gy (SF2) or 3 Gy (SF3). **(D)** RBE values calculated as the ratios of the alpha parameters of survival curves.

**Figure 3:**
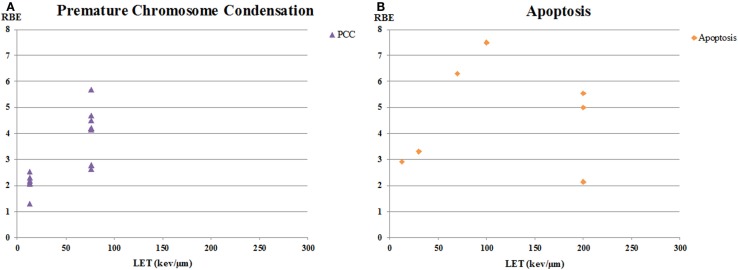
**RBE versus LET for human tumor cell lines for chromatin breaks and apoptosis**. **(A)** is from data on unrejoined chromatin breaks in cells after premature chromosome condensation (PCC). Residual unrejoined chromatin breaks were detected using Giemsa staining in cells after chromatin condensation. **(B)** is RBE values for apoptosis.

Endpoint is one of the major factors, which affects the values of RBE (7, 54, 55, 92). The RBE data as a function of LET sorted by endpoint are shown in Figures 2 and 3. All papers included in Figure 2 presented RBE values for colony formation after exposure to a range of single doses. Within a total of 363 RBE values, 295 values in 31 papers were calculated using an isoeffect dose of 10% survival (D10). The other values that were calculated included D0, D30, D50, D75, ratio of alpha parameters, or isodose effectiveness. The RBE values for D10 ranged from 1.03 to 4.99, showing the “classic” increase in RBE with LET followed by a decrease at higher LET (22) although the range in RBE values at any given LET is substantial in many cases. The RBE values based on D0, D30, D50, and D75 also showed considerable variation at any given LET, but, as expected, there was a trend for higher RBE values at higher levels of survival (22). Some of the highest RBE values were derived using the alpha ratio; this, too, is consistent with higher RBEs at higher survival, since alpha ratios would tend to be derived based on high survival data.

The other endpoint that tends to show high RBE values is apoptosis (Figure 3B). This is consistent with the observations that most solid tumor cell lines are resistant to X-ray-induced apoptosis (93) and that apoptosis may be characterized by the alpha-component of the cell survival curve [reviewed, e.g., in Ref. (94)]. In a recent review on proton radiobiology, Tommasino and Durante (95) pointed out that there is a general tendency for an increased apoptotic response with increasing LET and that tumor cells resistant to photon-induced apoptosis may have apoptosis triggered by an alternative pathway by protons, a suggestion that could likely extend to heavier charged particles. However, it should also be pointed out that several groups, including Brown and colleagues (96), have demonstrated that apoptosis induction can be markedly affected by tumor cell genetics and the overall level of cell killing as determined in a clonogenic assay *in vitro* may not correlate well with apoptosis induction [also reviewed in Ref. (94)].

Two papers reported RBE values calculated for residual unrepaired chromatin breaks using premature chromosome condensation (PCC) (Figure 3A), with the paper by Suzuki et al. using primary cells obtained by biopsy from patients (67, 72). Authors of both studies noted the good correlation between their data on residual chromatin breaks as measured using the PCC technique and colony formation, and concluded that the PCC technique was a potential predictive assay of tumor response to ion therapy. Information on correlation of chromatin breaks using PCC with DNA repair protein foci formation and/or FA/BRCA pathway status, as discussed above for potential use with proton therapy patients, would be helpful for assessment of possible predictive assays.

#### Ion

**Figure 4:**
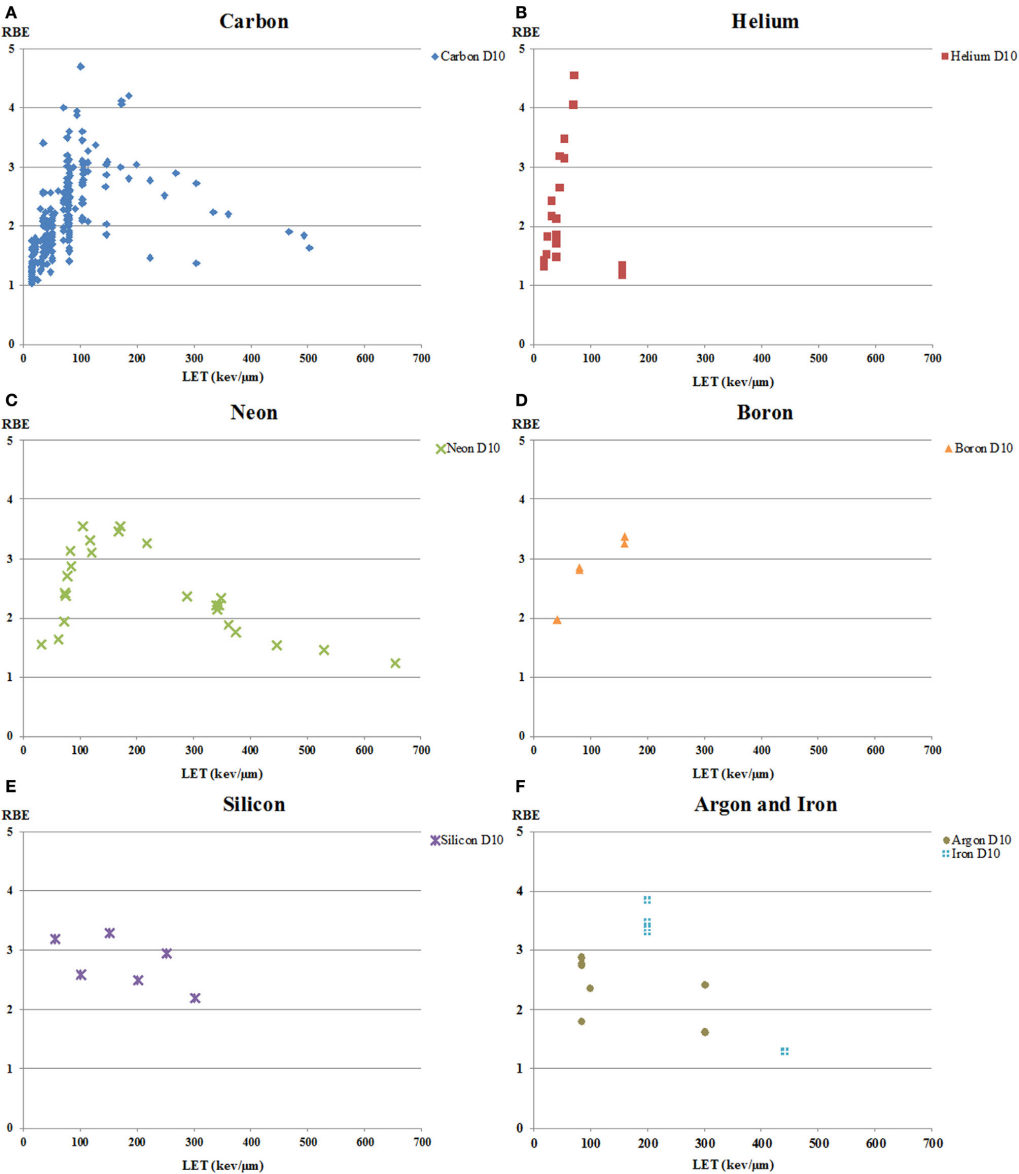
**RBE at 10% survival (D10) versus LET for human tumor cell lines exposed to various charged particles heavier than protons**. RBE values derived at 10% survival from clonogenic survival curves from all available literature are shown as a function of LET for human tumor cells exposed to **(A)** carbon ions; **(B)** helium ions; **(C)** neon ions; **(D)** boron ions; **(E)** silicon ions; and **(F)** argon and iron ions. The RBE values showed substantial variation at any given LET, independent of ion species, but in all cases the RBE increased with LET to a maximum, then decreased at high-LET levels.

The data on RBE values calculated using D10 and sorted by ions are shown in Figure 4. A total of 29 papers reported 247 RBE values for carbon-ion beam, whereas there were 21 RBE values for helium ions in 3 papers, 24 values for neon ion in 2 papers, 6 values for boron ions in 1 paper, 6 values for silicon beam in 2 papers, 5 values for iron beam in 3 papers, 2 values for nitrogen beam in one paper, and 3 values for argon beam in 2 papers. The RBE values showed substantial variation at any given LET, independent of ion species used, but in all cases the RBE increased with LET to a maximum then decreased at high-LET levels. It is well known that the RBE values of carbon ions peak around an LET of 100 keV/μm (7, 54, 55, 92). The other ion beams had peaks between LETs of 100 and 200 keV/μm, with a trend toward a maximum at higher LET with heavier ions.

Furusawa et al. (59) exposed human salivary gland tumor cells to carbon, neon, and helium ion beams and calculated the RBE values of each beam. They showed that the RBE values for helium ions were higher than those for the other ions, which seems unexpected. This finding deserves more investigation as there is some interest in development of helium ion beams for cancer therapy since they have less lateral dose than protons (i.e., a better dose distribution) (97), which might make their use particularly relevant in children. Furthermore, in their report, Furusawa et al. show that the peaks of the RBE values shifted to higher LET values with increasing atomic number, an observation that had been made earlier on the basis of work by a number of authors [e.g., Ref. (11, 98, 99)] as reviewed by Skarsgard (2). Such findings deserve emphasis as they highlight the fact that LET is not adequate as the sole descriptor of energy deposition in cells and tissues, but that ion track structure, the nanometer scale distribution of energy, must be considered when evaluating biological effects. In this context, it is interesting to note that NASA’s model for calculating risk of radiation-induced cancer from space radiation takes into account track structure of heavy ions rather than simply LET (100). In a clinical context in heavy ion therapy, the LEM, which is used for RBE prediction, also provides particle species and LET-specific RBE values that are then propagated, using a treatment planning system, to a representative RBE value at each position in the irradiated field (9, 101), a process needed because ion fragmentation produces a mixed radiation field.

With regard to ions, it is worth pointing out that we did not include data with oxygen ions in Figure 4 because we found only one study using oxygen ions, and that work used only a single LET (87). That work reported that for four human hepatocellular carcinoma cell lines irradiated in the SOPB of oxygen ions with a mean energy of 154 MeV/u (LET of 146 keV/μm), the clonogenic RBE_10_ values ranged from 1.9 to 3.1, with the values not being significantly different from those obtained in the same study using 130 MeV/u carbon ions (LET of 112 keV/μm). However, this study is noteworthy because of the current interest in using oxygen ions, with their lower OER, in treating tumors with large hypoxic fractions (102).

#### Type of Tumor Cells

**Figure 5:**
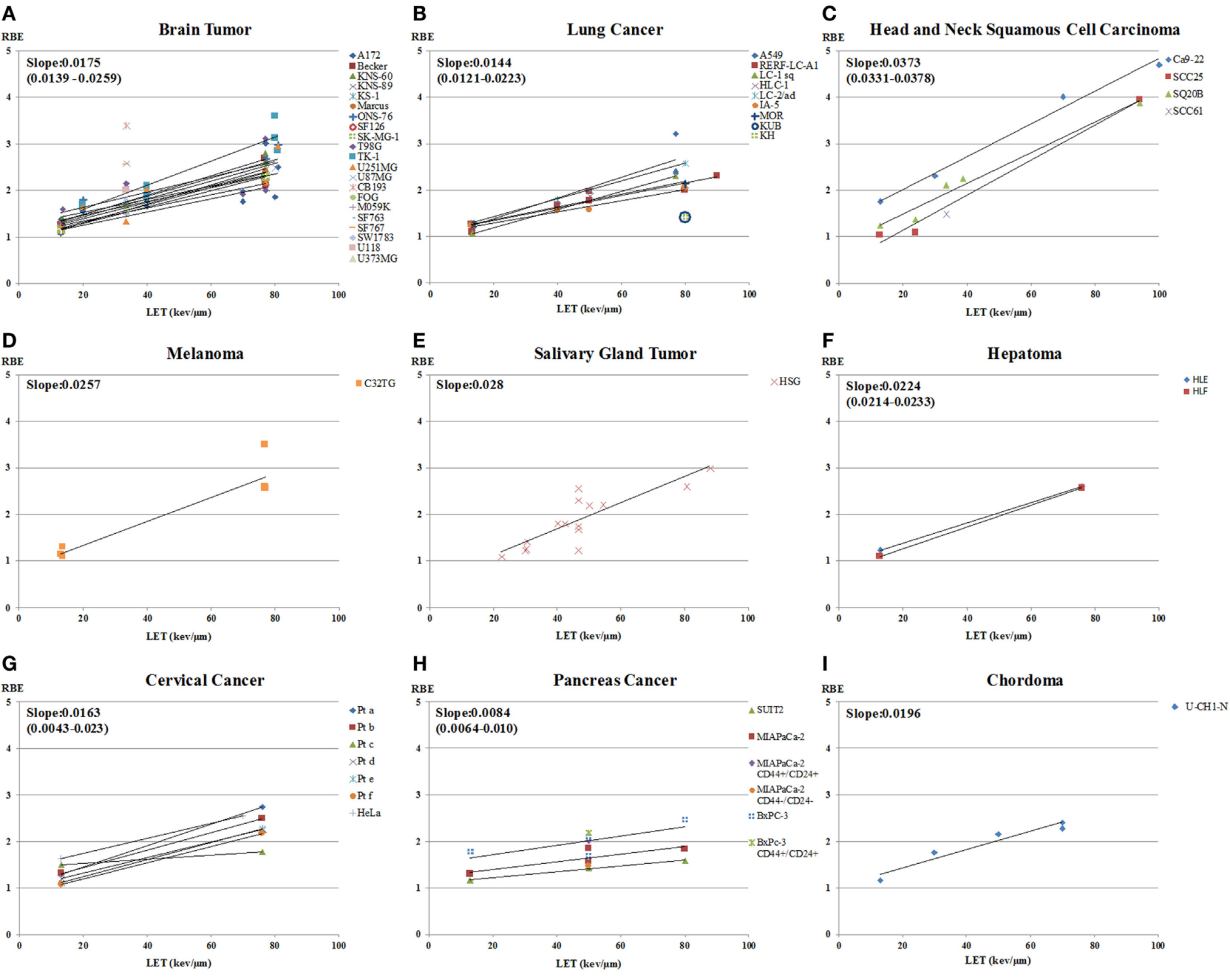
**RBE at 10% survival versus LET for cells from various types of human tumors exposed to carbon ions**. RBE values as a function of LET for carbon-ion beam only, calculated using D10 and sorted by tumor type, are shown. Tumor types included are: **(A)** brain tumor; **(B)** lung cancer; **(C)** head and neck squamous cell carcinoma; **(D)** melanoma; **(E)** salivary gland tumor; **(F)** hepatoma; **(G)** cervical cancer; **(H)** pancreatic cancer and **(I)** chordoma. The graphs include data only for LET < 100 keV/μm. The number of data points, or cell lines, varies greatly with tumor type. The slopes of the RBE versus LET curves are calculated for each cell line and tumor type.

**Figure 6:**
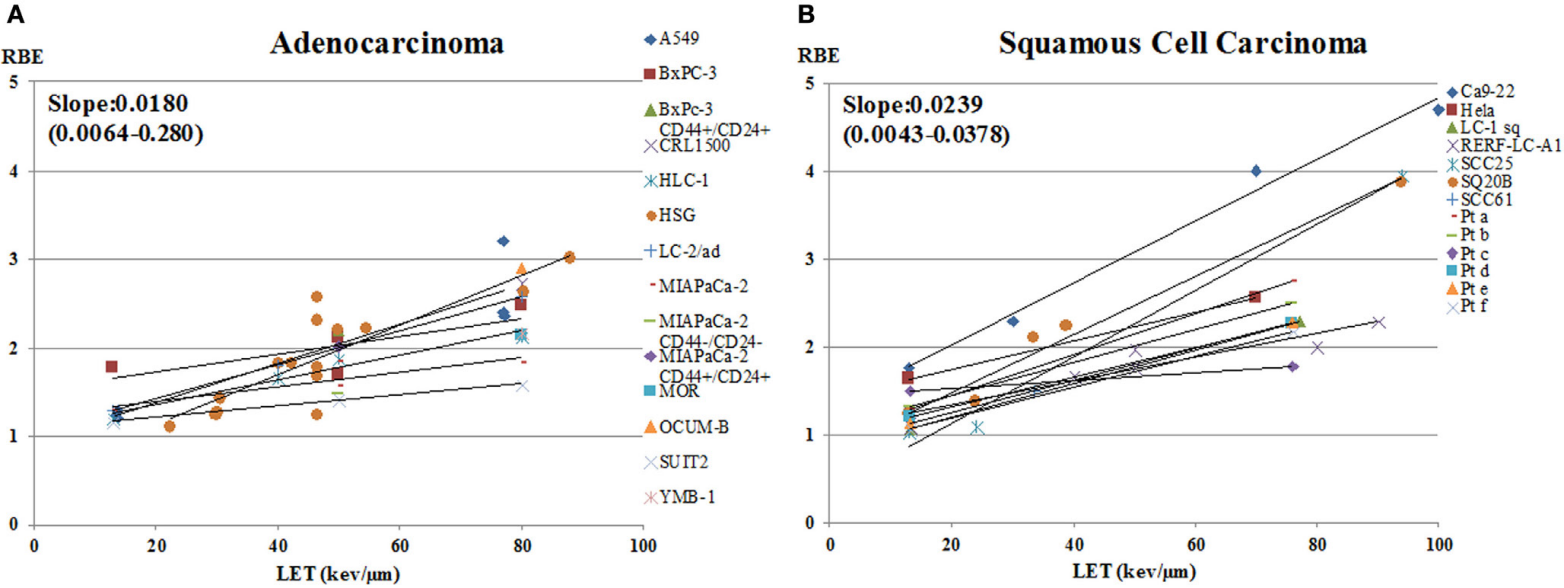
**RBE at 10% survival versus LET for cells from human adenocarcinomas and squamous cell carcinomas exposed to carbon ions**. RBE values as a function of LET for carbon ion beam only, calculated using D10 for **(A)** adenocarcinomas and **(B)** squamous cell carcinomas. The graphs show data only for LET < 100 keV/μm. The slopes of the curves are shown for each cell line.

The data on RBE values as a function of LET for carbon-ion beam only, calculated using D10 and sorted by tumor type, are shown in Figure 5. Figure 6 shows a subset of the data separated out by adenocarcinoma and squamous cell carcinoma. The graphs show data only for LET < 100 keV/μm. The number of data points, or cell lines, varies greatly with tumor type. Generally, the brain tumors (composite slope of 0.018) and adenocarcinomas (composite slope of 0.018) appear to have lower slopes for the RBE versus LET curves than do squamous cell carcinomas (composite slope of 0.024 or higher). It should be noted, however, that data from Suzuki et al. (71) for cervical cancer included in the squamous cell carcinoma graph were derived from primary cultured cells from biopsies from patients, the only data from primary cultures included in this analysis. These primary culture data appear to have lower slopes than the other squamous cell data, although it should also be noted that the steeper slopes for the established squamous carcinoma cell lines are determined by only four data points at high-LET values. Thus, it is not possible to determine whether there is a systematic difference between primary squamous cell cultures compared to established tumor cell lines or between squamous cell carcinomas and adenocarcinomas. It is not clear from clinical data with carbon ions whether a difference exists between sensitivity of squamous tumors and adenocarcinomas, suggesting an area for further *in vitro*, *in vivo*, and/or clinical study. For comparison, it can be pointed out that in a similar analysis approach, Ando (54) found that the RBE versus LET plot for cultured human fibroblasts had a slope of 0.027, which the author noted was steeper than the composite slope for the human tumor data he analyzed.

The RBE values for the pancreas cancer cells are the lowest in all the data (64). The slope of the graph of pancreas is 0.0084, which is gentler than the others. This might suggest that pancreatic cancer would not be a good candidate for carbon-ion therapy, yet clinical trials of carbon ions for pancreas cancer in Japan have shown promising results (4, 103). The clinical results may reflect properties of the human tumors *in situ*, such as high hypoxia, radioresistance (high cancer stem cell component?), and anatomic location, that might not be evident in studies of isolated tumor cells.

It is noteworthy that there are few tumor cell data on RBE values with charged particles for prostate cancer or bone and soft tissue cancers, which are the two cancer types with the most patients treated to-date with carbon ions at NIRS in Japan (103). Furthermore, we found no experimental RBE data for human cell lines of mucosal malignant melanoma, adenoid cystic carcinoma, or rectal carcinoma, which are all being treated with carbon ions at NIRS with favorable outcomes (103).

D10 has been used as the parameter for calculating RBE values in this analysis by tumor type (Figures 5 and 6) because that is the parameter most frequently reported in the literature. However, the use of D10 may have minimized the ability to see differences between tumor cell types, resulting in the relatively similar values of the slopes of the RBE versus LET curves for the various tumor cells. Generally, inherent photon radiosensitivity differences between cell types become most evident at high and low cell survival levels, and it has long been recognized that RBE values are larger at high survival levels than at low ones because of the “shoulder” on photon survival curves (22). For example, this is consistent with the data shown in Figure 2 where RBEs based on alpha ratio (generally reflecting high survival, low dose results) tend to be higher than those based on D10. Since it has been shown that photon dose–response curves for different tumor cell types have significant differences [e.g., Ref. (104, 105)], one might expect that the RBE versus LET curves would also differ in a manner consistent with the photon sensitivity. The finding here (Figures 5 and 6) that the differences seem small may reflect the use of the less discriminating parameter, D10. If sufficient data existed to do this analysis with a parameter more weighted toward lower or, especially, higher survival levels, e.g., alpha ratio, greater differences in dependence of RBE on LET for various cell types might be seen.

### Role of Genetic Background of Tumor Cells in Response to Charged Particles

In light of the proton data, discussed above, indicating a correlation between cell lines with higher proton RBE values and defects in DNA repair, specifically in HR repair, we wondered whether the same finding would extend to heavier ions, notably carbon ions. Although the literature data on RBEs for human tumor cell lines shows substantial variations at any given LET, even just for carbon ions (Figures 2–6), we could not find any information in the literature on possible DNA repair deficiencies, particularly in HR repair, for the cell lines with the highest RBEs after carbon-ion irradiation, e.g., TK-1 brain tumor, Ca9–22 gingival squamous cell carcinoma, SQ20B head-and-neck cancer. Therefore, experiments to ascertain carbon RBE values for human tumor cell lines known to be defective in the FA/BRCA DNA repair pathway are warranted. Furthermore, both the proton data of Liu et al. (44) and the carbon-ion data of Suzuki et al. (70) on residual unrepaired DNA damage (assays of 53BP1 foci and DNA damage revealed by PCC, respectively) suggest that such assays may be useful biodosimeters to select patients for charged particle therapy.

What would be the clinical application of increased tumor RBE values in subsets of patients? Identifying patients with proton- and/or heavy ion-sensitive tumors may allow us to: (a) de-escalate the physical dose of charged particles if normal tissue damage is a particular concern; (b) select patients for proton or heavy ion treatment slots who would have not otherwise had the opportunity to be treated with such radiations, thereby increasing the odds of local tumor control; or (c) biologically optimize tumor-directed therapy, for example, by employing intensity-modulated ion therapy algorithms to superimpose an LET increase on the already pre-existing RBE advantage, thereby further improving local tumor control. Because RBE values tend to increase with increasing fractionation sensitivity of tumors (i.e., decreasing alpha/beta values) (42), there exists additional opportunity to improve the outcome of ion beam therapy in tumors with low alpha/beta values, such as prostate or breast cancer. However, this approach will require better knowledge of the inter-tumoral variation of alpha/beta values and the development of predictive biomarkers to identify appropriate tumors.

## Sensitivity of Cancer Stem-Like Cells to Charged Particles

In recent years, considerable interest has developed in the possibility that cancer stem-like cells (CSCs) in human tumors could be major contributors to resistance of tumors to conventional photon radiotherapy (RT) (106–108). However, intriguing data also suggest that the presence of CSCs might be overcome by carbon-ion therapy (89, 109). In this section, we discuss such a potential from a radiobiological perspective.

Cancer stem-like cells, also called cancer-initiating cells (CICs), are tumorigenic and have the potential to give rise to all cell types identified in hematological cancers and in several types of solid tumors (110). CSCs are regarded as “roots of cancer,” analogous to normal stem cells in hierarchical tissues, although the origin of CSCs is still not clear and various theories have been proposed to explain their origin (111). It is believed that tumor growth is driven by a discrete subpopulation of CSCs that are defined by their capacity for self-renewal and their ability to generate heterogeneous lineages of cancer cells (110). The CSCs can survive and usually persist in tumors for a substantial length of time as a distinct population and can eventually cause cancer recurrence after treatment and tumor metastasis. It seems reasonable to suggest that cancer cure can be achieved only if this population is eliminated.

There is growing evidence that CSCs are inherently resistant to conventional fractionated RT. This radioresistant phenomenon of CSCs has been described within the framework of the four Rs of radiobiology: (i) repair, (ii) redistribution, (iii) reoxygenation, and (iv) repopulation (112).

(i) Regarding DNA repair, CSCs exhibit fewer DNA DSBs after exposure to ionizing radiation than non-tumorigenic cancer cells, which has been correlated with efficient DNA repair machinery due to constitutive hyperphosphorylation of the DNA checkpoint kinases Chk1 and Chk2 (106).
(ii) Regarding redistribution, quiescent or slowly cycling cells, normal or cancer stem cells, generally are radioresistant, although dose fractionation can cause redistribution of radioresistant S-phase cells into a more sensitive phase of the cell cycle. If this happens only in tumor cells, it could result in a therapeutic benefit for slowly cycling normal cells, sparing late responding normal tissues during fractionation. However, if tumors also have a significant proportion of CSCs that are slowly cycling, any benefit from redistribution may not apply (112).
(iii) Regarding reoxygenation, if the niche in which CSCs reside is hypoxic, during radiation fractionation the quiescent CSCs may be exposed to increasing oxygen levels causing increasing radiosensitivity due to transition of cells into an activated, proliferative state. It appears that in some cases, the CSC niche may be in perivascular regions (113, 114) where they may be exposed to rapidly changing cycles of hypoxia-reoxygenation (112). During reoxygenation, the cells would become more radiosensitive, and reoxygenation triggers metabolic processes that generate damaging reactive oxygen species (ROS). However, CSCs manifest enhanced protection against ROS (107, 108). It was reported that expression of the CSC marker CD44, in particular that of a variant isoform (CD44v), contributes to ROS defense by promoting the synthesis of glutathione (GSH), a primary intracellular antioxidant radical scavenger (115). Hence, the roles of hypoxia, reoxygenation, and ROS defenses in CSCs appear quite complex, and more research is required to elucidate their roles in radiation response.
(iv) Regarding repopulation, it was reported that developmental signaling pathways, such as TGF-β, Notch, Wnt/B-catenin, and Sonic hedgehog pathways greatly contribute to maintenance of CSCs, as they do with normal tissue stem cells (112). Intrinsic inter-conversion and dynamic equilibrium between CSCs and non-stem cancer cells (NSCCs) exist under normal and irradiation conditions, and TGF-β might have important roles in the equilibrium (116).

In addition to the four Rs of radiobiology, it has been shown that CSCs can acquire radioresistance through activation of anti-apoptotic Bcl-2 (117) and serine/threonine protein kinase B (PKB, also known as AKT) survival signaling (118, 119). Hence, there is substantial reason to believe that CSCs are a radiation-resistant cell population in at least some tumors exposed to photon irradiation.

**Figure 7:**
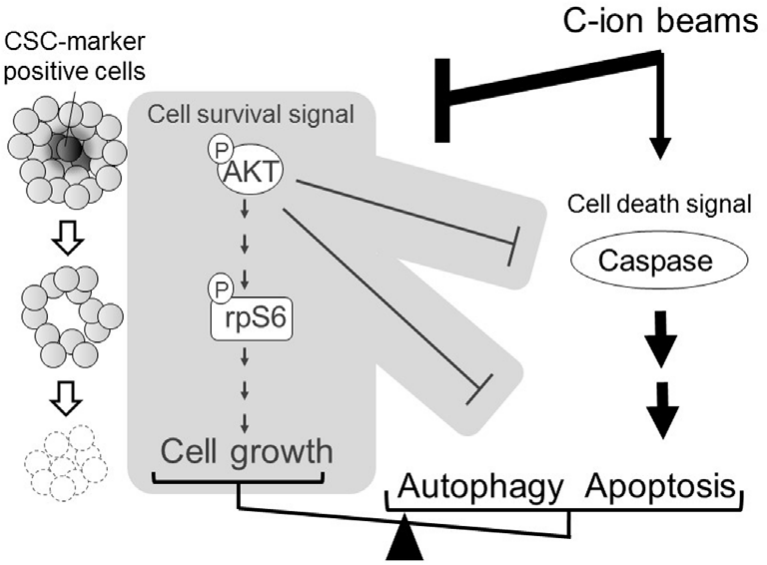
**A model for carbon ion-induced apoptosis and autophagy through the enhancement of death signals and the depression of survival signals**. The model is based on AKT survival signaling as shown in our work (126). An arrow “→” indicates enhancement; a sidewise “⊣” indicates depression.

On the other hand, intriguing studies have reported that CSCs may be more effectively killed by carbon ions compared to photons in colon and pancreas cancers both *in vitro* and *in vivo* (89, 109, 120), and CSCs from colon and breast cancers may be more efficiently eliminated by proton irradiation than photon treatment, at least *in vitro* (121, 122). One or more of several processes may explain the observations that ion beams have biological advantages for killing CSCs compared to photons. These include the diminished capacity for NHEJ repair, which may play an important role in the quiescent G0 cell cycle phase, after heavy ion exposure (39); a decreased OER with heavy ions (59, 123), and an efficacy in dealing with radioresistant tumor cells (*TP53*-mutated and BCL2-overexpressing cells) (124) compared with results produced by photon beams. We demonstrated that heavy ion beams depress AKT-related survival signaling (125). Therefore, we speculate that heavy ion beams may target CSCs *via* depression of AKT survival signaling. Indeed, we demonstrated that the population of CSCs is only slightly increased or unchanged after carbon-ion irradiation because carbon ions may simultaneously kill CSCs and non-CSCs, while X-rays have less effect on CSCs than on the bulk cancer cells (126). These results suggest that carbon ions may enhance apoptosis and autophagy through activation of death signaling and may target CSCs *via* the depression of AKT survival signaling (Figure 7). However, it should be noted that the observations of CSCs being preferentially more sensitive to charged particles is not universal as it has been reported that head-and-neck cancer CSCs are resistant to both photon and carbon-ion irradiation (127). Clearly, more detailed studies are necessary, for example, using tumor samples from carbon- and photon-irradiated patients, to understand the potential significant therapeutic benefit of heavier charged particles on CSCs. It is also worth investigating whether, or how, the enhanced DNA repair advantages in CSCs might relate to the potential for development of biomarkers based on residual DNA damage for identifying patients whose cancers might be treated more efficaciously using charged particles, as discussed in the section above.

## Sensitivity of Hypoxic Tumor Cells to Charged Particles

A long-recognized property of tumors is their development of hypoxic regions. It has also been documented, for many years, that hypoxic cells are resistant to photons, but that resistance is reduced when hypoxic cells are irradiated with higher LET particles (22). Suit et al. (3) postulated that the potential gain from high-LET radiations in the clinic may be due principally to the lower OER (ratio of doses for a given endpoint in hypoxic to well-aerated cells). This section discusses the reduced hypoxic protection with carbon-ion therapy and how that might be exploited in cancer therapy.

Cellular sensitivity to low-LET radiations (photons, clinical energy protons) depends on the degree of hypoxia at the time of irradiation, increasing in a sigmoid fashion from an OER = 1.0 (no difference between anoxic and well-aerated cells) at very low oxygen levels to a maximum (OER ~ 3.0) usually obtained by about 2–3% oxygen (22, 128). Sensitivity also depends on the duration of exposure to the hypoxic conditions. There are two distinct mechanisms that promote oxygen deficiency in tumor cells; each exposes cells to different periods of hypoxia. Acute or perfusion-limited hypoxia is caused by poorly formed or dysfunctional vasculature that can cause transient closing of blood vessels that deprives the surrounding cells of an appropriate oxygen supply (129). On the other hand, in chronic or diffusion-limited hypoxia, the imbalance of oxygen supply and consumption in actively proliferating tumor cells causes cells far from blood vessels to experience a deficiency in oxygenation for long periods of time (130). Historically, most studies of hypoxic radioresistance have dealt with chronic hypoxia, but experiments investigating the influence of acute and chronic oxygenation conditions on cell response have shown increased radioresistance for the acute case (131–133). Ma and colleagues demonstrated that for both X-ray and carbon-ion irradiation, cells under acute anoxia were more radioresistant than those under chronic anoxia, whereas cells subjected to acute and chronic hypoxia (0.5% O_2_) exhibited no significant difference in sensitivity (131, 132). They argued that prolonged exposure to anoxia induced a breakdown in cellular energy metabolism, which led to delays in cell cycle progression. They found that cells were arrested in the G1 phase of the cell cycle with a significant decrease in the number of active S-phase cells after 24 h of hypoxia. However, abrupt changes in the oxygenation status did not result in changes in the cell cycle distribution. The energy deficiency of cells also has been associated with the reduction of DNA damage repair (133). Therefore, chronically hypoxic cells were found to be more vulnerable to radiation damage.

**Figure 8:**
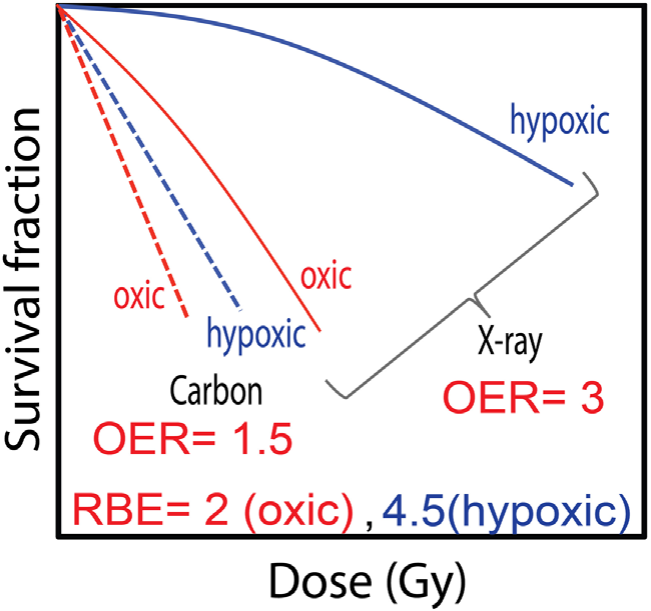
**The dependence of survival curves on oxygen concentration typically observed after exposure to X-rays and carbon ions**. The importance of the oxygen effect is reduced with high-LET carbon-ion irradiation as is apparent in the small separation of the survival curves compared to that seen with X-rays. The large difference between the cell response in air and hypoxia for X-rays results in a *RBE*_hypoxic_ that is greater than *RBE*_oxic_.

The poor performance of photons in curing hypoxic tumor cells has prompted researchers to turn to high-LET radiation, such as ion beams that have lower OERs [reviewed, e.g., in Ref. (22, 128, 134, 135)]. Radiation damage from low-LET beams is mostly mediated by free radicals (indirect effects), i.e., secondary electrons generated from the ionizations interact with molecules, such as water, to produce free radicals which in turn damage the DNA. In contrast to their low-LET counterparts, the contribution of radiation damage by direct ionizations in DNA is higher for high-LET beams. Here, the secondary electrons directly interact with the critical target, thus producing, at least in part, different damage. Hence, the oxygen effect can be explained, at least in part, by differences in induction and repair of DNA damage. Hirayama et al. reported that the rejoining kinetics of DNA DSBs incurred from carbon-ion irradiation were the same for cells in oxic and hypoxic conditions (136). This led them to postulate that DNA DSBs produced by carbon ions are the same for the two oxygenation conditions. However, their results for X-ray irradiation showed a dependence of the repair dynamics on the oxygen level, with DSBs generated under oxic conditions rejoined more efficiently than those produced under hypoxia. They postulated that this resulted from different mechanisms for DNA damage depending on oxygenation, namely, that in the presence of oxygen, oxygen-reacting radicals could cause additional DNA DSBs but in hypoxia more damage is produced by direct ionizations or by radicals irrelevant to oxygen. Furthermore, the repair times were longer after carbon-ion irradiation and more unrepaired DNA DSBs remained after 5 h while for X-rays almost all DSBs were efficiently rejoined. This can be explained by the high ionization density generated along the track of heavy charged particles that produces complex DNA damages, making repair more difficult. Therefore, the OER decreases with increasing LET values, with the OER of carbon ions about half that with X-rays. Typical survival curves obtained using carbon-ion and X-ray irradiation under oxic and hypoxic conditions are illustrated in Figure 8. The difference with oxygenation status is diminished with the high-LET carbon ions and the survival curves tend to converge. By contrast, the larger variation in the cell response seen for X-ray irradiation is reflected by the higher OER value. A consequence of the enhanced radioresistance observed in X-ray survival curves under hypoxia is that *RBE*_hypoxic_ generally exceeds *RBE*_oxic_. *In vitro* studies have also shown that OER approaches unity at dose-averaged LETs of ~300 keV/μm (59, 134). Oxygen ions, with their high-LET values within therapeutic fields, might be advantageous for tumors with significant hypoxic fractions. Scifoni et al. (135) compared computed OER values in a tumor irradiated with oxygen or carbon ions, and showed that, assuming the same dose in the entrance region, there was a dramatic decrease in OER for the oxygen ions.

The advantage of high-LET carbon ions over photons in treating tumor hypoxia has been confirmed in the clinical setting by Nakano et al. (137). They measured the intratumor oxygen partial pressure of uterine cervical cancer patients prior to and at the fifth day of treatment with either photons or carbon ions using a polarographic electrode. The 4-year local control rates were found to be independent of the oxygenation condition for carbon-ion treatment, whereas the control rate for photon therapy of patients with high *p*O_2_ status was more than twice that with low *p*O_2_.

It has been suggested that further improvements in treatment outcome with carbon therapy can be achieved by considering the time course of reoxygenation of hypoxic areas in the tumor. According to Antonovic et al. (138), the number of fractions and the dose per fraction for carbon therapy can be optimized by taking into account the effect of local oxygenation changes on tumor control probability. In the future, more detailed studies are necessary to take into account the OER and rates of reoxygenation in treatment planning for carbon-ion RT, as are underway (134, 135).

## Dose Fractionation with Charged Particles

Fractionated irradiation is a valuable tool in conventional RT to reduce early and late effects in normal tissue by allowing repair of sublethal damage or increase tumor response due to reoxygenation of a hypoxic tumor. The linear quadratic (LQ) model describes cell killing using single-hit and double-hit components (22). The shape of the curve is determined by:

(1) $$\text{SF}(\text{D})={\text{e}}^{-\left(\alpha \text{D}+\beta {\text{D}}^{2}\right)}.$$

The α parameter describes the linear component of the curve, while the β component describes the quadratic portion of the curve. The α/β ratio, the point at which linear cell killing is equivalent to quadratic cell killing, is an important parameter used to model cell killing by radiation. Presently, this ratio is used as a staple for predicting the clinical effects in response to RT despite various limitations. A high α/β ratio, seen in many human tumors, suggests a predominance of the α component, implying a decreased response to fractionation and, therefore, clinical benefit from hypofractionation (decreased number of fractions of larger dose per fraction). A lower α/β ratio is usually associated with late responding normal tissue and is the basis for the therapeutic gain achieved using hyperfractionation (increased number of fractions of small dose per fraction), which allows for greater repair/recovery of normal tissues (139). However, some human tumors, e.g., prostate cancer, melanoma, and some sarcomas, may have α/β values similar to late responding tissues (140–142).

In image-guided RT, intensity-modulated RT, and X-ray stereotactic body RT (SBRT), there are tendencies to reduce the number of fractions and increase the dose per fraction (i.e., hypofractionation) (143, 144). With carbon-ion RT, superiority of the physical dose distribution can lead to a reduction in the number of fractions (145), allowing hypofractionation. There are few relevant experimental data using human tumor cells on hypofractionation effects with high-LET charged particles. Experiments involving high-LET fast neutron beams demonstrated that increasing the dose per fraction tended to decrease the RBE for both tumor and normal tissues (146). However, the dose-dependent decrease in the RBE for the tumor was less pronounced than that for normal tissues, such as skin and lung (147). These experiments led to the assumption that the therapeutic gain of carbon-ion RT would increase when the dose per fraction increased. This assumption was confirmed in animal experiments that compared RBE for carbon ions between tumor and skin (148). In additional studies with high-LET radiation, RBE depends on dose and dose per fraction: dose-dependent decrease of RBE was reported after fast neutrons to normal skin, intestine, growing cartilage, and hematopoietic tissues (149), and after Ne-ions to the skin of mice and hamsters (150). The change in dose dependence is caused by the higher α/β ratio of target cells after high-LET radiation than after photons (151).

**Figure 9:**
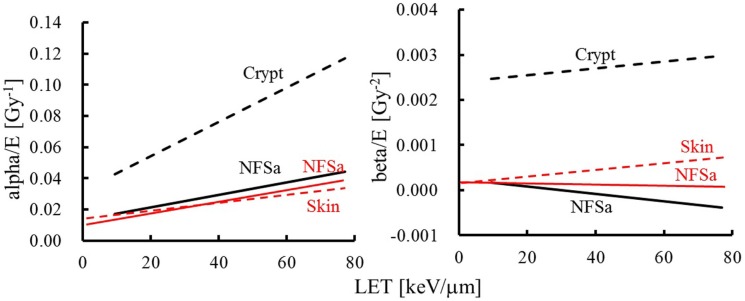
**α and β terms for mouse normal tissues and tumors plotted against LET**. The comparison of survival parameters of Ando et al. (red) (148) and our study (153) using crypt survival and tumor growth delay assays (black). The solid lines show NFSa tumor and broken lines show the normal tissues (crypt or skin).

The value of the α term increases with increasing LET in both tumor and normal tissue, while the issue of whether the value of the β term changes with LET remains controversial (148, 152). In our study (153) by evaluating the therapeutic gain of carbon-ion fractionation using intestinal crypt survival and tumor growth delay (TGD) assays, the values of the α and β terms for the mouse fibrosarcoma (NFSa) tumor are close to those reported by Ando et al. (148), while those for normal tissues are different (Figure 9). In addition, the LET-dependent increase (e.g., slope of the regression line) of the α term for NFSa is similar to that for human salivary gland tumor cells (148, 154). LET-dependent increase of α terms for crypt is greater than that for the early skin reaction after daily fractionated doses to leg skin (148), whereas it is similar to that for the late skin reaction after 4-h interval fractionations to foot skin (154). These results indicate that therapeutic gain for carbon-ion RT depends on the normal tissue and fractionation schedule. Further studies with mouse skin and rat spinal cord where the normal tissues were exposed to varying numbers of fractions and doses per fraction of γ-rays and carbon ions have shown that the magnitude of damage repair depends on both the number of fractions and the size of dose per fraction for high-LET radiation (155, 156). It was concluded that repair of radiation injury is much reduced with dose per fraction, especially with 125 keV/μm carbon ions. Unfortunately, few studies of fractionation effects with carbon ions have been performed with tumors, especially human tumors.

As discussed above, hypoxia is one of the main factors reducing local control in some solid tumors, and fractionation in RT may have an advantage because of reoxygenation of the hypoxic areas. It has been reported that reoxygenation in several tumors irradiated with carbon ions occurs earlier than that in those irradiated with photons (157, 158). Reoxygenation in the NFSa fibrosarcoma was observed at 4 days, 1 day, and within 0.5 days after irradiation with photons, low-LET carbon ions (14 and 18 keV/μm) and high-LET carbon ions (43, 58, and 74 keV/μm), respectively (157). Thus, short-term fractionated irradiation with carbon ions may be effective in the treatment of tumors, at least in part, because of altered reoxygenation.

The clinical RBE is replaced by an LET-dependent RBE for *in vitro* cell killing data determined in single-dose experiments and is employed to design the SOBP and in the Japanese treatment planning system for carbon-ion RT (159, 160). A question remains as to whether the biological effects with fractionated doses are also uniform within the SOBP. Uzawa et al. evaluated uniformity of a new ridge filter that was designed based on α and β values for various LETs to cause mouse foot skin reaction by carbon-ion fractionated irradiation (154). The physical dose distribution of the new ridge filter was almost identical to the ridge filter designed based on *in vitro* cell kill. While the LQ model is useful for conversion between relatively low radiation doses as used in conventional RT, it has been suggested that it is not applicable to higher fractional doses or smaller fraction numbers (6, 161). It has been questioned whether the LQ model is applicable to hypofractionated carbon-ion RT. For establishment of the optimal fractionation strategy in carbon-ion RT, applicability of the LQ model should be investigated in future studies.

With photon RT, the rapidly expanding use of hypofractionation even to the extreme of single fractions as used in stereotactic radiosurgery (SRS) and SBRT has lead to recent discussion about whether “new” biology should be advanced to explain the greater than expected anti-tumor efficacy of some hypofractionation regimens. Some have proposed that consideration of only the clonogenic survival of only the tumor cells is not sufficient to account for the observed responses [e.g., Ref. (162, 163)], although not all agree [e.g., Ref. (164–166)]. Brown et al. reviewed the clinical data for early-stage NSCLC and suggested that radiobiological modeling with the LQ model is adequate to explain the efficacy of SRS and SBRT (166). Fowler showed the potential advantages of hypofractionation for prostate cancer by using the LQ model and concluded that use of the LQ model can yield consistent results, for example, the remarkable agreement for tumor effects of some of the best schedules in regular use (167). It is likely that the same considerations apply to carbon-ion therapy, although few data exist, especially for human tumors in experimental situations. Here, we briefly review some aspects that may pertain.

In some situations, vascular damage may be a dominant pathway for tumor suppression. Irradiation of human tumor xenografts or rodent tumors with 5–10 Gy in a single dose causes relatively mild vascular damages. On the other hand, numerous studies with experimental tumors indicate that irradiation with doses higher than 10 Gy in a single fraction or 20–60 Gy in limited numbers of fractions causes severe vascular damage, including endothelial cell apoptosis, leading to the deterioration of the intratumor microenvironment and indirect death of tumor cells (163, 168, 169). Little is known about the vascular changes in human tumors treated with high-dose hypofractionation, particularly with heavy ions, but experimentation is indicated to address whether radiation-induced vascular damage and the resulting indirect death of tumor cells may play important roles in the response of tumors to high-dose hypofractionation with charged particles.

In addition to potential vascular effects, it has been suggested that high-dose irradiation evokes immune reactions and thereby eradicates tumor cells that escaped radiation-induced death (170, 171). In support of such notion, a recent report showed that ablative RT dramatically increased T-cell priming in lymphoid tissues, leading to reduction/eradication of the primary tumor or distant metastasis in a CD8^+^ T-cell dependent fashion (170). Several studies have shown that carbon ions induce anti-tumor immunity (172–176), although the effects of high-LET radiation on immune function have not been studied in detail. Hence, enhanced immune reactions might be involved in the response of tumors to high-dose hypofractionation, especially with charged particles (177).

It is also noteworthy that, unlike photon irradiation, particle irradiation may suppress the metastatic potential of cancer cells (172, 178), and a recent paper has shown that there is a decrease in metastasis with decreasing fraction number of carbon ions (179). Clearly, further studies are warranted to gain better insights into the effects of high-dose hypofractionation with heavy ions on tumor vasculature, immune system, and metastasis, and how such biology might impact human tumor RBE values and therapeutic gain.

## Conclusion

In a recent review of charged particle therapy, Loeffler and Durante (4) stated that “Considering the current uncertainties in clinical results [with charged particles] and the difficulties in performing clinical trials, research in physics and radiobiology should reduce the cost/benefit ratio.” In this review, we have focused on discussion of selected aspects of radiobiological data with human tumors exposed to protons and heavier charged particles, raising specific instances where further laboratory research may contribute to improving particle therapy. With increasing understanding of the genetic heterogeneity in human tumors, particularly with regard to alterations in DNA repair pathways, a fruitful research area appears to be elucidation of DNA repair pathways selectively involved in repair of the unique clustered DNA damages caused by charged particles. With increases in such knowledge, the differences can be exploited to identify patients who may be better treated with particles because of characteristics of their tumors and to develop novel pharmacologic approaches that capitalize on the differences in DNA damage and repair. Another area ripe for charged particle biology study with implications to clinical advances is in cancer stem cells. The intriguing observations that cancer stem cells from human tumors may be more effectively killed by carbon ions than by photons begs for further study on mechanisms involved – altered DNA repair? location in a hypoxic niche? – and consideration of how to exploit such a difference to the advantage of ion therapy. Finally, the biology underlying the notable clinical effectiveness of high dose, hypofractionated charged particles, which may be explained by radiosensitivity of tumor cells themselves at high doses or may involve vasculature and/or immune system responses, requires further elucidation.

This article has focused on data from *in vitro* studies of human tumor cells, for reasons described in the Section “Introduction” and recognizing that there are limitations when applying *in vitro* findings to the *in vivo* and clinical situations. However, it is also clear that because of the stochastic natures of radiation-induced cell killing and tumor cure and the, albeit simplistic, relationship of the two endpoints *via* TCP = e^−(SF × M)^ (where TCP is tumor control probability, SF is surviving fraction, and M is number of clonogens), understanding effects of radiations of varying qualities on the tumor cells themselves can be informative. The questions and issues raised herein require follow-up *in vivo* studies leading to transfer of knowledge to the clinic, but guidance from the *in vitro* work, e.g., on use of DNA damage assays and exploiting DNA repair as biomarkers for patient selection or using *in vitro* survival α/β information to help guide design of hypofractionation protocols *in vivo* and in the clinic, is critical.

## Author Contributions

KH, HK, TK, AP, YY, QL, HW, and AT contributed to the conception, drafting, and revising of the review article.

## Conflict of Interest Statement

The authors declare that preparation of this article was conducted in the absence of any commercial or financial relationships that could be construed as a potential conflict of interest.
